# Supplementary material for: Simultaneous Occurrence of Multiple Neoplasms in Children with Cancer Predisposition Syndromes: Collaborating with Abnormal Genes
Source: Genes (Basel). 2023 Aug 24;14(9):1670. doi: 10.3390/genes14091670 (PMC10530991; doi:10.3390/genes14091670)
Supplement: Supplementary file 1 [file genes-14-01670-s001.zip › genes-2505782-supplementary.pdf]

Table S1. A detailed list of genes in the NGS oncology panel.

AIP	*ALK*	*ANKRD26*	*APC*	*ATM*
AXIN2	*BAP1*	*BARD1*	*BLM*	*BRAF*
BRCA1	*BRCA2*	*BRIP1*	*CBL*	*CD70*
CDC73	*CDH1*	*CDK4*	*CDKN1B*	*CDKN1C*
DDB2	*DDX41*	*DICER1*	*DKC1*	*EGFR*
ELANE	*EPCAM*	*ERCC1*	*ERCC2*	*ERCC3*
ERCC4	*ERCC5*	*ETV6*	*EXQ1*	*EXT1*
EXT2	*E2H2*	*FANCA*	*FANCB*	*FANCC*
FANCD2	*FANCE*	*FANCF*	*FANCG*	*FANCI*
FANCL	*FANCM*	*FH*	*FLCN*	*GATA2*
GPC3	*GREM1*	*HNF1A*	*HRAS*	*IKZF1*
KIT	*KRAS*	*LZTR1*	*MAP2K1*	*MAP2K2*
MAX	*MEN1*	*MET*	*MITF*	*MLH1*
MLH3	*MRE11A*	*MSH2*	*MSH6*	*MUTYH*
NBN	*NF1*	*NF2*	*NRAS*	*NSD1*
NSUN2	*NTHL1*	*PALB2*	*PAX5*	*PDGFRA*
PHOX2B	*PMS1*	*PMS2*	*POLD1*	*POLE*
POT1	*PPM1D*	*PRF1*	*PRKAR1A*	*PTCH1*
PTEN	*PTPN11*	*RAD50*	*RAD51C*	*RAF1*
RET	*RIT1*	*RUNX1*	*SDHA*	*SDHAF2*
SDHB	*SDHC*	*SDHD*	*SHOC2*	*SLX4*
SMAD4	*SMARCA4*	*SMARCB1*	*SOS1*	*SOS2*
SPRED1	*STK11*	*SUFU*	*TERC*	*TERT*
TINF2	*TMEM127*	*TP53*	*TSC1*	*TSC2*
VHL	*WT1*	*XPA*	*XPC*	*XRCC2*
